# Cornel Iridoid Glycoside Ameliorated Alzheimer's Disease-Like Pathologies and Necroptosis through RIPK1/MLKL Pathway in Young and Aged SAMP8 Mice

**DOI:** 10.1155/2021/9920962

**Published:** 2021-08-23

**Authors:** Denglei Ma, Yanzheng Li, Yanqiu Zhu, Weipeng Wei, Li Zhang, Yali Li, Lin Li, Lan Zhang

**Affiliations:** ^1^Department of Pharmacy, Xuanwu Hospital of Capital Medical University, National Center for Neurological Disorders, National Clinical Research Center for Geriatric Diseases, Beijing Engineering Research Center for Nervous System Drugs, Beijing Institute for Brain Disorders, Key Laboratory for Neurodegenerative Diseases of Ministry of Education, Beijing 100053, China; ^2^Hebei Medical University, Shijiazhuang, Hebei 050017, China

## Abstract

**Background:**

Aging is an important risk factor for sporadic Alzheimer's disease (AD) and other neurodegenerative diseases. Senescence-accelerated mouse-prone 8 (SAMP8) is used as an animal model for brain aging and sporadic AD research studies. The aim of the current study was to investigate the pharmacological effects of cornel iridoid glycoside (CIG), an active ingredient of *Cornus officinalis*, on AD-type pathological changes in young and aged SAMP8 mice.

**Methods:**

Locomotor activity test was used to detect the aging process of SAMP8 mice. Nissl staining and immunohistochemical staining were applied to detect neurons and myelin basic protein-labelled myelin sheath. Western blotting was used to detect the expression levels of related proteins of synapse, APP processing, and necroptosis.

**Results:**

The results showed that SAMP8 mice at the age of 6 and 14 months exhibited lower locomotor activity, age-related neuronal loss, demyelination, synaptic damage, and APP amyloidogenic processing. In addition, the increased levels of receptor-interacting protein kinase-1 (RIPK1), mixed lineage kinase domain-like protein (MLKL), and p-MLKL indicating necroptosis were found in the brain of SAMP8 mice. Intragastric administration of CIG for 2 months improved locomotor activity; alleviated neuronal loss and demyelination; increased the expression of synaptophysin, postsynaptic density protein 95, and AMPA receptor subunit 1; elevated the levels of soluble APP*α* fragment and disintegrin and metalloproteinase 10 (ADAM10); and decreased the levels of RIPK1, *p*-MLKL, and MLKL in the brain of young and aged SAMP8 mice.

**Conclusion:**

This study denoted that CIG might be a potential drug for aging-related neurodegenerative diseases such as AD.

## 1. Introduction

Aging is closely related to decline of learning and memory, with a higher incidence of neurodegenerative diseases, including sporadic AD [1, 2]. Senescence-accelerated mouse prone 8 (SAMP8) is a mouse stain with accelerated senescence status developed from AKR/J series [3] and has been used as an animal model for brain aging and AD [4]. Numerous articles have demonstrated that SAMP8 mice display advancing aging status and share common characteristics with the aged and AD patients, including age-related deteriorative cognition and behavioral alteration [4, 5], neuropathological phenotypes such as neuron loss and synaptic plasticity impairment [6, 7], hyperphosphorylation of tau forming neurofibrillary tangles [8], APP amyloidogenic processing [9], and other pathological features in age-related neurodegeneration [10]. Senescence-accelerated mouse-resistant 1 (SAMR1) exhibits normal phenotypes and is used as non-age-accelerated control of SAMP8 mice [11].

*Cornus officinalis* Sieb. et Zucc is a traditional herbal medicine and widely applied to treat age-related diseases and dementia in China. Cornel iridoid glycoside (CIG) is the main effective ingredient of *Cornus officinalis*. Morroniside and loganin are the major components of CIG. It has been reported that morroniside and loganin exhibit the antioxidative [12] and antiosteoporosis [13] activities and inhibit cholinesterase and *ß*-secretase activities *in vitro* [14], as well as antidiabetic effect *in vivo* [15]. In our previous studies, CIG showed protective pharmacological effects against focal cerebral ischemia [16, 17] and traumatic brain injury by inhibiting inflammation and apoptosis [18]. We recently found that CIG suppressed tau hyperphosphorylation and aggregation through activating protein phosphatase 2 A in a P301L mutant tau transgenic mouse model [19–21].

As aging is one of the key risk factors of AD, we studied the pharmacological effects of CIG using SAMP8 mice. In our previous article, we found SAMP8 mice showed cognitive impairments and senescent status, and CIG treatment reversed these changes at different ages [22]. However, the effects and mechanisms of CIG on the AD pathologies on SAMP8 mice remain unclear.

Necroptosis is a form of programmed necrotic cell death caused by many microenvironmental factors [23]. Recent studies found that necroptosis is involved in the normal aging and several neurodegenerative disorders, such as AD [24–26]. Whether necroptosis plays a role in the pathologies in SAMP8 mice and the intervention effects of CIG remains unclear. In the current study, we investigated the effects of CIG on neuronal loss, demyelination, synaptic damage, APP amyloidogenic processing, and necroptosis in the brain of young and aged SAMP8 mice.

## 2. Materials and Methods

### 2.1. Drugs

Cornel iridoid glycoside (CIG) was extracted from the sarcocarp of *Cornus officinalis* Sieb. et Zucc as described in our previous paper [16]. *Cornus officinalis* Sieb. et Zucc was purchased from Beijing Tongrentang Company (Beijing, China). Morroniside accounted for 67%, and loganin, 33%. As oxiracetam has been approved as a nootropic agent to treat patients with AD clinically [27], oxiracetam is used as a positive control drug in the present study.

### 2.2. Animals

Male SAMP8 and SAMR1 mice were obtained from the First Affiliated Hospital, Tianjin University of Traditional Chinese Medicine (Tianjin, China). All mice were housed under a normal light-dark (12 h/12 h) cycle and standard temperature conditions (22 ± 2°C), with free access to food and clean water. All mice were habituated for 7 days before starting the experiment.

### 2.3. Animal Grouping and Treatment

Two different ages of SAMR1/SAMP8 mice were applied and allocated to two experiment tranches as previously reported [22]. (1) Young SAMP8 mice at 4-month-old received treatments of three doses CIG (50, 100, and 200 mg/kg/d), saline (as model group), or oxiracetam (as positive control drug, 360 mg/kg/d) for 2 months; same age SAMR1 mice were treated with saline or 100 mg/kg/d CIG; *n* = 15 per group. (2) Aged SAMP8 mice at 12-month-old received saline (*n* = 18) or CIG (200 mg/kg/d; *n* = 15); SAMR1 mice (*n* = 12) received normal saline for 2 months.

The dosages of CIG were chosen based on our previous studies in mice (Ma et al.), and the dosage of oxiracetam was converted from human clinical dosage. CIG and oxiracetam were dissolved in normal saline, intragastrically administered to mice once a day and lasted for 2 months.

### 2.4. Locomotor Activity Test

The locomotor activity assay consisted of a large cabinet with 4 dark cages (20 cm × 35 cm × 18 cm). On the testing day, mice were placed individually in cages, and left for 8 min without any disturbance. Total spontaneous activity counts within the last 5 min were recorded by the infrared sensors in the cages.

### 2.5. Tissue Collection

For immunohistochemical analysis, mice were perfused transcardially with 4% paraformaldehyde after being anesthetized by intraperitoneal injection of 1.25% avertin (0.2 ml/10 g body weight) (Sigma, USA). The brain was removed, postfixed, and then dehydrated in 15∼30% sucrose/0.1 M PBS. Brain tissues were cut into series horizontal sections with 30 *μ*m thick in a cryostat slicer after being frozen in isopentane (620E, Thermo fisher Scientific, USA).

For western blotting, brain tissues (4 mice per group) were homogenized in lysis buffer (50 mM tris-HCl, 0.1% SDS, 150 mM NaCl, 1% Nonidet P-40, 2 mM EGTA, 0.5% sodium deoxycholate) with phosphatase/protease inhibitor cocktail (Thermo fisher Scientific, USA). Homogenates were centrifuged at 12,000*g* for 20 min at 4°C. Supernatants were collected and boiled for 5 min. Consequently, RC-DC Protein Assay Kit (Bio-Rad Laboratories, USA) was applied to determine protein concentrations.

### 2.6. Nissl and Immunohistochemical Staining

For Nissl staining, dehydrated brain sections were stained with 0.1% cresyl violet acetate (SigmaAldrich, St. Louis, MO, USA) for 20 min, rinsed in distilled water three times (2 min), differentiated in 95% ethanol with acetic acid (1 min), dehydrated with alcohol, and cover-slipped with neutral balsam (ZSGB-Bio, Beijing, China).

Three brain slices in each mouse were subjected to immunohistochemical staining of NeuN and MBP. Endogenous peroxidase activity was blocked by exposing to 3% H_2_O_2_ for 15 min and then sealed in 10% serum at 37°C for 1 h. The sections were then incubated with the primary antibodies (Table 1) at 4°C. After being washed, sections were then incubated with goat anti-rabbit/mouse nonbiotin detection system (PV9002/9001, ZSBiO, Beijing, China), and immune complexes were visualized by a DAB substrate kit (ZSBiO, Beijing, China).

Pictures were photographed under Olympus microscope and analyzed using Image-Pro plus 5.0 software (Media Cybernetics, Inc., Bethesda, USA). For comparison of the number of neurons between groups, signals were extracted from images by using the color threshold function with identical settings in Image-Pro plus 5.0 software. Three slices per mouse were analyzed to get an average value. Quantification of the number of neurons was achieved and analyzed by experimenter blinded to the groups.

### 2.7. Western Blotting

Proteins were loaded and separated on 10% SDS-PAGE gel and transferred onto polyvinylidene fluoride (PVDF) membranes (Millipore, USA). Membranes were then blocked by 5% nonfat milk in TBST buffer (tris-buffered saline-Tween 20, consisting of 10 mM Tris-HCl, 100 mM NaCl, and 0.05% Tween-20) and incubated with primary antibodies (see Table 1). On the second day, after incubation with a horseradish peroxidase-conjugated anti-rabbit or anti-mouse IgG secondary antibody (1 : 2000, Cell Signaling Technology, USA), immune complex was detected by ECL detection reagent for Western blotting (Immobilon™ Western Chemiluminescent HRP Substrate, Millipore, USA). Band intensity was analyzed using TINA (Raytest Isotopenme Bgerate 190 GmbH, Straubenhardt, Germany).

### 2.8. Statistical Analysis

All data were provided as mean ± S.E.M. (standard error of mean). Data were analyzed using one-way ANOVA followed by *Tukey's post hoc* test to determine statistical significance among groups. *P* < 0.05 was regarded as statistically significant. Graphs were plotted in Prism version 5.0 software (GraphPad Software Inc., USA).

## 3. Results

### 3.1. CIG Alleviated Aging Process of Young and Aged SAMP8 Mice in Locomotor Activity Test

**Figure 1:**
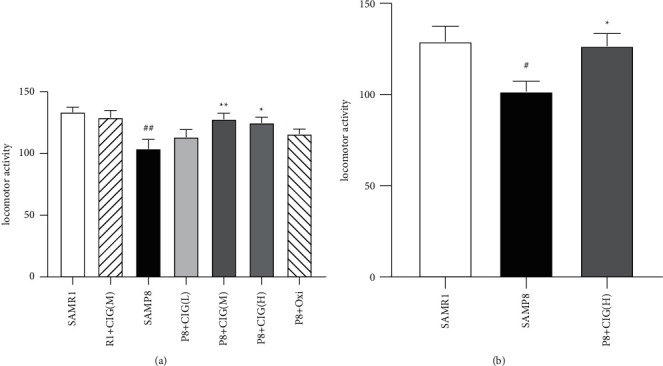
Effects of CIG on locomotor activity of SAMP8 mice at young and aged SAMP8 mice. (a) Quantitative analysis of total times in locomotor activity of 6-month-old SAMP8 mice; (b) quantitative analysis of total times in locomotor activity of 14-month-old SAMP8 mice. Data are expressed as the mean ± S.E.M., *n* = 10∼15 each group. ^#^*P* < 0.05, ^##^*P* < 0.01, SAMP8 model group *vs* SAMR1 control group; ^*∗*^*P* < 0.05 and ^*∗∗*^*P* < 0.01, drug-treated SAMP8 groups *vs*. SAMP8 model group. SAMR1, senescence accelerated mouse/resistant 1; R1 + CIG (M), SAMR1 mice treated with CIG at 100 mg/kg; SAMP8, senescence accelerated mouse/prone 8; P8 + CIG (L), SAMP8 mice treated with CIG at 50 mg/kg; P8 + CIG (M), SAMP8 mice treated with CIG at 100 mg/kg; P8 + CIG (L), SAMP8 mice treated with CIG at 200 mg/kg; P8 + oxi, and SAMP8 mice treated with oxiracetam at 360 mg/kg.

Spontaneous locomotor activity represents the aging process and depression of SAMP8 mice. In the present study, young and aged SAMP8 mice exhibited lower spontaneous locomotor activity compared with age-matched SAMR1 mice (*P* < 0.01, *P* < 0.05; Figure 1). CIG treatment significantly increased the spontaneous locomotor activity of SAMP8 mice (*P* < 0.01 and *P* < 0.05; Figure 1).

### 3.2. CIG Alleviated Neurons Loss and Demyelination in the Brain of Young and Aged SAMP8 Mice

**Figure 2:**
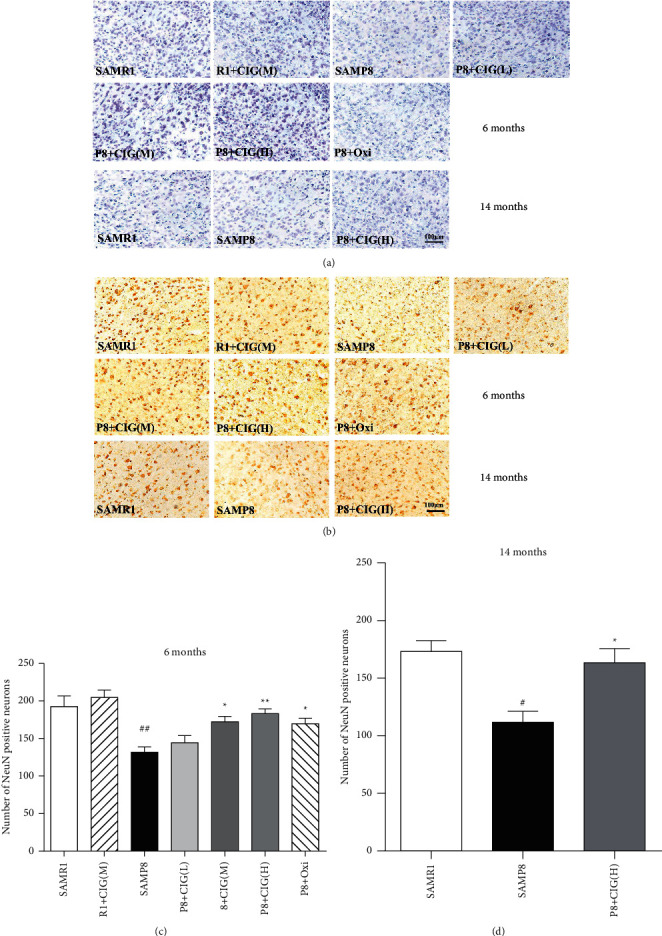
Effects of CIG on neuronal loss in the cerebral cortex of SAMP8 mice at young and aged SAMP8 mice. (a) Representative images of Nissl staining in the cerebral cortex of mice at 6 and 14 months of age. (b) Representative images of immunohistochemical staining for NeuN-labelled neurons in the cerebral cortex of mice at 6 and 14 months of age; scale bar = 100 *μ*m. (c) Quantitative analysis of the number of NeuN-labelled neurons in the cerebral cortex of mice at 6 months of age and (d) at 14 months of age. Data are expressed as the mean ± S.E.M., *n* = 3 each group. ^#^*P* < 0.05, ^##^*P* < 0.01, and SAMP8 model group *vs*. SAMR1 control group; ^*∗*^*P* < 0.05, ^*∗∗*^*P* < 0.01, drug-treated SAMP8 groups *vs.* SAMP8 model group. NeuN; neuronal nuclei antigen.

The neurons in the brain of mice were detected by NeuN immunohistochemical staining. The results indicated a notable loss of neurons in the cerebral cortex of SAMP8 at the ages of 6 and 14 months compared with age-matched control SAMR1 mice (*P* < 0.01, *P* < 0.05). Intragastric administration of CIG and oxiracetam for 2 months significantly increased the number of neurons in the cerebral cortex of young and aged SAMP8 mice (*P* < 0.01 and *P* < 0.05; Figure 2).

**Figure 3:**
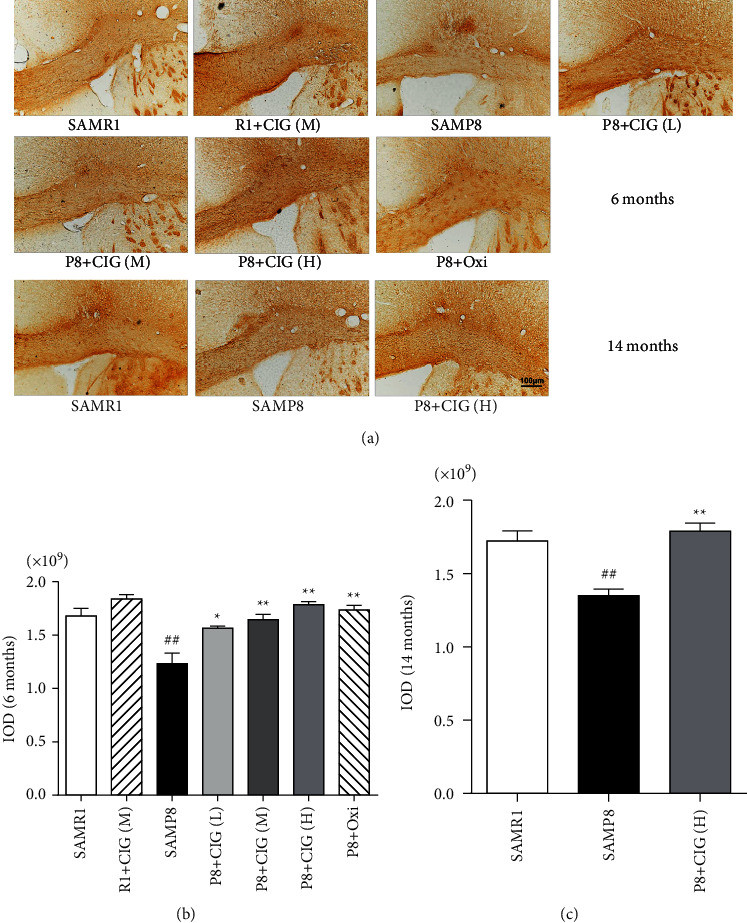
Effects of CIG on demyelination in the corpus callosum of SAMP8 mice at young and aged SAMP8 mice. (a) Representative images of myelin basic protein (MBP) immunohistochemistry in the corpus callosum of SAMP8 mice at young and aged SAMP8 mice; scale bar = 100 *μ*m. (b) Quantitative analysis of integrated optical density (IOD) for MBP immunohistochemistry at 6 months of age and (c) at 14 months of age. Data are expressed as the mean ± S.E.M., *n* = 3 each group. ^#^*P* < 0.05 and ^##^*P* < 0.01, SAMP8 model group *vs.* SAMR1 control group; ^*∗*^*P* < 0.05 and ^*∗∗*^*P* < 0.01, drug-treated SAMP8 groups *vs.* SAMP8 model group. IOD, integrated optical density.

Myelin basic protein (MBP), the main protein of the myelin sheath, is used to represent mature oligodendrocyte and the integrity of myelin. In the present study, the immunohistochemistry results showed that the expression of MBP was evidently declined in the corpus callosum of young and aged SAMP8 rats (*P* < 0.05; Figure 3). However, treatment with CIG (100 and 200 mg/kg) significantly elevated the expression of MBP in young and aged SAMP8 mice (*P* < 0.05; Figure 3). These results demonstrated that CIG reduced demyelination of young and aged SAMP8 mice.

### 3.3. CIG Increased the Expression of Synaptic-Related Proteins in the Hippocampus of Young and Aged SAMP8 Mice

**Figure 4:**
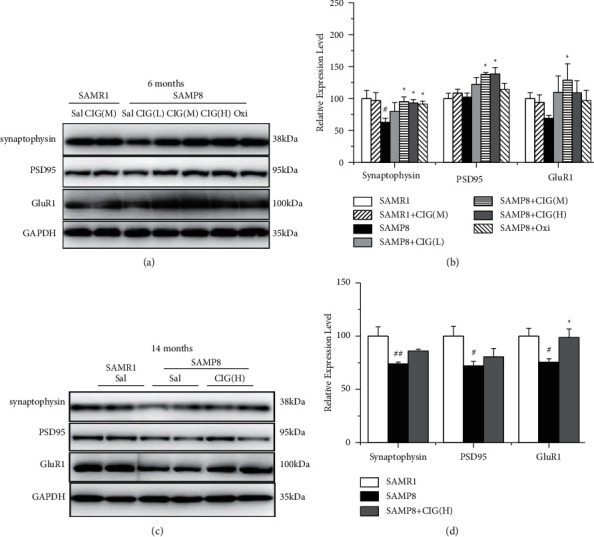
Effects of CIG on the expression of synaptic plasticity-related proteins in the hippocampus of young and aged SAMP8 mice (western blotting). (a, b) Representative images and quantitative analysis of the expression of synaptophysin, PSD95, and GluR1 in the hippocampus of SAMP8 mice at 6 months of age and (c, d) at 14 months of age. GAPDH served as an internal loading control, and the relative intensity in the SAMR1 control group was set as 100%. Data are expressed as the mean ± S.E.M., *n* = 4 each group. ^#^*P* < 0.05 and ^##^*P* < 0.01, SAMP8 model group *vs* SAMR1 control group; ^*∗*^*P* < 0.05 and ^*∗∗*^*P* < 0.01, drug-treated SAMP8 groups *vs.* SAMP8 model group. Sal, normal saline (vehicle); PSD95, postsynaptic density protein 95; GluR1, AMPA receptor subunit 1.

The normal integration of synaptic proteins and glutamate receptors at the synapse determines the synaptic plasticity, which is closely associated with cognitive functions [28]. Synaptophysin is a presynaptic protein, and postsynaptic density protein 95 (PSD95) is mainly expressed in the postsynaptic area [29]. In the present study, western blotting results showed that the expression of synaptophysin decreased in the hippocampus of young and aged SAMP8 model mice compared with SAMR1 (*P* < 0.05 and *P* < 0.01). CIG and oxiracetam treatment significantly elevated the levels of synaptophysin in young SAMP8 mice (*P* < 0.05; Figure 4). Moreover, the expression of PSD95 declined in the hippocampus of aged SAMP8 model mice compared with SAMR1 (*P* < 0.05); CIG treatment significantly increased the levels of PSD95 in young SAMP8 mice (*P* < 0.05; Figure 4).

GluR1, a subunit of *a*-amino-3-hydroxy-5-methyl-4-isoxazolepropionic acid receptors (AMPA) receptor, plays important roles in synaptic transmissions and long-term potentiation (LTP) [30]. In the present study, western blotting results showed GluR1 expression obviously decreased in the hippocampus of aged SAMP8 model mice compared with the SAMR1 control group (*P* < 0.05); CIG treatment significantly increased the levels of GluR1 in young and aged SAMP8 mice (*P* < 0.05; Figure 4).

### 3.4. CIG Promoted APP Nonamyloidogenic Processing in the Cerebral Cortex of SAMP8 Mice at Different Ages

**Figure 5:**
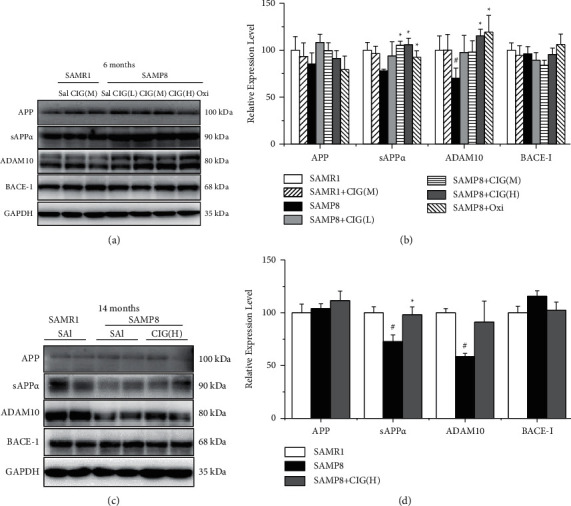
Effects of CIG on the expression of APP-related proteins in the cerebral cortex of SAMP8 mice at different ages (western blotting). (a, b) Representative images and quantitative analysis of the expression of APP, sAPP*α*, ADAM10, and BACE-1 in the cerebral cortex of mice at 6 months of age and (c, d) at 14 months of age. GAPDH served as an internal loading control and the relative intensity in the SAMR1 control group was set as 100%. Data are expressed as the mean ± S.E.M., *n* = 4 each group. ^#^*P* < 0.05 and ^##^*P* < 0.01, SAMP8 model group *vs.* SAMR1 control group; ^*∗*^*P* < 0.05 and ^*∗∗*^*P* < 0.01, drug-treated SAMP8 groups *vs.* SAMP8 model group. APP, *β*-amyloid precursor protein; sAPP*α*, soluble APP*α* fragment; ADAM10, a disintegrin and metalloproteinase 10 (*α*-secretase); BACE-1, *β*-site APP cleaving enzyme (*β*-secretase).

*β*-Amyloid precursor protein (APP) can be cleaved by ADAM10 (*α*-secretase) and releases a neuroprotective fragment, which is considered as APP nonamyloidogenic processing [31]. In the current study, SAMP8 model mice showed lower protein levels of ADAM10 at 6 and 14 months of age (*P* < 0.05) and sAPP*α* at 14 months of age in the cerebral cortex compared with the SAMR1 control group (*P* < 0.05, Figure 5). CIG treatment evidently increased the levels of ADAM10 and sAPP*α* in young SAMP8 mice (*P* < 0.05) and also significantly elevated the level of sAPP*α* in aged SAMP8 mice (*P* < 0.05; Figure 5). Oxiracetam obviously elevated the expression of ADAM10 and sAPP*α* in young SAMP8 mice (*P* < 0.05; Figure 5). There was no significant difference in the expression levels of full-length APP and BACE-1 among all groups of young and aged SAMP8 mice (Figure 5).

### 3.5. CIG Inhibited RIPK1/MLKL Pathway in the Cerebral Cortex of SAMP8 Mice

**Figure 6:**
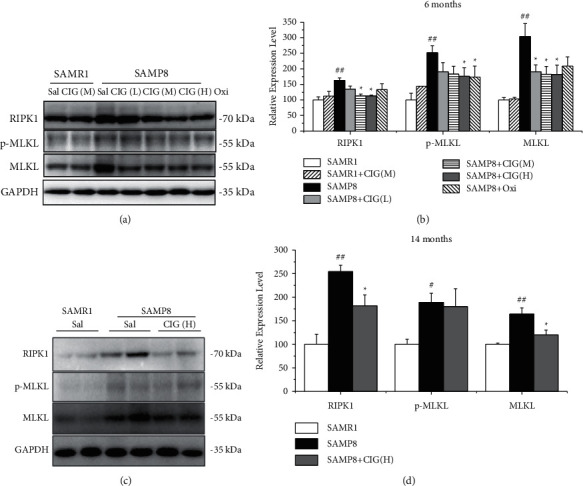
Effects of CIG on the RIPK1/MLKL pathway in the cerebral cortex of young and aged SAMP8 mice (western blotting). (a, b) Representative images and quantitative analysis of the expression of RIPK1, phosphorylated MLKL, and total MLKL in the cerebral cortex of mice at 6 months of age and (c, d) at 14 months of age. GAPDH served as an internal loading control, and the relative intensity in the SAMR1 control group was set as 100%. Data are expressed as the mean ± S.E.M., *n* = 4 each group. ^#^*P* < 0.05 and ^##^*P* < 0.01: SAMP8 model group *vs.* SAMR1 control group; ^*∗*^*P* < 0.05 and ^*∗∗*^*P* < 0.01: drug-treated SAMP8 groups *vs*. SAMP8 model group. RIPK1, receptor-interacting protein kinase-1; MLKL, mixed lineage kinase domain-like protein; p-MLKL, phosphorylated MLKL at Ser345.

Receptor-interacting protein kinase-1 (RIPK1) and mixed lineage kinase domain-like protein (MLKL) are the key elements that mediate necroptosis [24]. In the present study, we detected the changes of RIPK1 and MLKL in the brain of SAMP8 mice using western blotting. The results showed that the expression of RIPK1 markedly increased in the cerebral cortex of young and aged SAMP8 model mice compared with SAMR1 control mice (*P* < 0.01), and CIG treatment evidently decreased the level of RIPK1 in young and aged SAMP8 mice (*P* < 0.05; Figure 6). Moreover, the expression of phosphorylated and total MLKL increased obviously in the cerebral cortex of SAMP8 model mice (*P* < 0.01 and *P* < 0.05). CIG treatment significantly reduced the levels of phosphorylated MLKL in the cerebral cortex of young SAMP8 mice (*P* < 0.05) and total MLKL in young and aged SAMP8 mice (*P* < 0.05; Figure 6). Oxiracetam markedly decreased the expression of phosphorylated MLKL in young SAMP8 mice (*P* < 0.05; Figure 6).

## 4. Discussion

The present study revealed that CIG treatment effectively improved locomotor activity, ameliorated neuronal loss and demyelination, increased synaptic proteins (synaptophysin, PSD95 and GluR1) in the brain of young and aged SAMP8 mice. Meanwhile, CIG also increased the APP nonamyloidogenic processing by increasing sAPP*α* and ADAM10. Moreover, CIG inhibited necroptosis through downregulating the RIPK1/MLKL pathway. As a positive control drug, oxiracetam increased the expression of MBP, synaptophysin, ADAM10, and sAPP*α* and decreased the level of phosphorylated MLKL in the brain of SAMP8 at 6 months of age. Compared with oxiracetam, CIG showed better effects on ameliorating AD-related pathologies.

From the age of 6 months onward, SAMP8 mice exhibit obvious and age-related A*β* deposition, a major pathogenesis and pathology of AD [9]. Pathogenic A*β* fragment is generated through amyloidogenic pathways of APP processing by *ß*-secretase (BACE1) and *γ*-secretase. In healthy brain, the major proteolytic way of APP is processed by *a*-secretase (ADAM10), which produces a soluble and nontoxic APP fragment (sAPP*α*) and a C-terminal fragment [32]. Consistent with former studies [33, 34], we found decreased levels of ADAM10 and sAPP*α* in the brain of SAMP8 mice at the ages of 6 and 14 months. Several studies have found that active compounds decrease brain A*β* accumulation and prolonged survival in SAMP8 mice via increasing the expression of ADAM10 [34, 35]. In the current study, CIG treatment obviously increased expression levels of ADAM10 and sAPP*α* in the brain of SAMP8 mice at 6 and 14 months of age, indicating CIG may inhibit the A*β* production via activating the nonamyloidogenic processing of APP.

Synapse is the basic element involved in normal neuronal interactions in the brain, and synaptic plasticity is the biological basis of learning and memory [28]. In synapse, *a*-amino-3-hydroxy-5-methyl-4-isoxazolepropionic acid receptors (AMPARs) belong to glutamate receptors involved in many forms of synaptic plasticity (including LTP) and excitatory neurotransmissions in the hippocampus [36]. Propagation of toxic proteins (such as A*β* and phosphorylated tau) through synapse and synaptic dysfunction appear to be important contributors to cognitive impairments, and therapies targeting these deficits show the potential to improve cognition in AD [37, 38]. Consistent with previous studies [7], we found obvious synaptic loss and decreased expression of synaptophysin, PSD-95, and GluR1 in the hippocampus of young and aged SAMP8 mice. CIG treatment significantly reversed these changes, suggesting that CIG may protect the normal synaptic transmission and cognitive impairment in SAMP8 mice.

RIPK1 and necroptosis are activated in AD brain, positively correlated with Braak stage, and inversely correlated with brain weight and cognitive scores [24, 39]. Lowering necroptosis activation by inhibiting RIPK1 was reported to reduce cell loss in a mouse model of AD [24]. RIPK1 is a key molecule to initiate necroptosis and interacts with RIPK3 into complex IIb [40]. And, MLKL, a pseudokinase, would be phosphorylated and inserted into the plasma membrane as oligomers, leading to the initiation of necroptosis [41, 42]. In the current study, expression levels of RIPK1, phosphorylated, and total MLKL were increased, indicating the activated necroptosis in the brain of young and aged SAMP8 mice. Treatment with CIG evidently reversed these changes in SAMP8 mice. It is suggested that CIG may inhibit the activation of RIPK1/MLKL pathway and necroptosis, which may explain CIG's protective effects on neuronal loss in the brain of SAMP8 mice.

Earlier interventions play an important role in delaying the onset of AD [43]. The learning and memory ability of SAMP8 mouse strain is impaired as early as 4 months of age, and the average lifespan of SAMP8 mice is 12–14 months old [7, 44]. In our study, we used two tranches of SAMP8 mice at 4- and 12 months old to represent the different stages of AD and investigated the effects of CIG intervention over different phases. In our previous article, CIG improved cognitive impairments in SAMP8 mice at earlier phases [22]. In the present article, CIG showed better effects on APP nonamyloidogenic processing, synaptic plasticity, and necroptosis at earlier stages, which may explain the effects of earlier intervention on cognitive impairments. Through our research on the different intervention effects of CIG on SAMP8 mice, we proposed a suggestion that earlier treatment of CIG might benefit the patients of AD or mild cognitive impairments (MCI).

## 5. Conclusion

In conclusion, the current study demonstrated that two-month treatment of CIG improved locomotor activity of SAMP8 mice, alleviated neuronal loss and demyelination, and enhanced synaptic transmission via increasing the levels of synaptophysin, PSD95, and GluR1 in the brain of SAMP8 mice at 6 and 14 months. Moreover, CIG promoted the APP nonamyloidogenic processing through increasing ADAM10 (*α*-secretase) and sAPP*α*. Additionally, CIG inhibited necroptosis through downregulating the RIPK1/MLKL pathway in the brain of SAMP8 mice. It is suggested that CIG may be a potential drug for aging-related neurodegenerative diseases such as AD. And, earlier treatment of CIG might show better effects on improving pathological changes and cognitive impairments for patients of AD or MCI.

## Figures and Tables

**Table 1:** Primary antibodies used in this study.

Antibody	Type	Species	Dilution	Company	Catalog	Use
NeuN	Mono-	M	1 : 200	Millipore	MAB377	IHC
MBP	Mono-	Rat	1 : 200	Millipore	MAB386	IHC
Synaptophysin	Poly-	R	1 : 1000	Sigma	SAB4502906	WB
PSD95	Poly-	R	1 : 1000	CST	3409	WB
GluR1	Poly-	R	1 : 1000	Abcam	ab31232	WB
GAPDH	Mono-	R	1 : 1000	CST	5174	WB
APP	Mono-	R	1 : 1000	CST	2452	WB
sAPP*α*	Poly-	R	1 : 1000	Covance	SIG-39139	WB
ADAM10	Poly-	R	1 : 1000	Sigma	A2726	WB
BACE-1	Poly-	R	1 : 1000	Abcam	ab2077	WB
RIPK1	Mono-	R	1 : 1000	CST	3493	WB
p-MLKL (Ser345)	Mono -	R	1 : 1000	CST	37333	WB
MLKL	Mono -	R	1 : 1000	CST	37705	WB

Mono-, monoclonal; poly-, polyclonal; M, mouse; R, rabbit; IHC, immunohistochemistry; WB, western blotting.

## Data Availability

The data used to support the findings of this study are available from the corresponding author upon request.
